# Study on Structural Design and Manufacturing of Sandwich Composite Floor for Automotive Structure

**DOI:** 10.3390/ma14071732

**Published:** 2021-04-01

**Authors:** Hoyeon Lee, Hyunbum Park

**Affiliations:** 1 JAEYI Corporation Co., Ltd, 26 Muyeok-ro, Gunsan 54002, Korea; librahyl@gmail.com; 2 School of Mechanical Convergence System Engineering, Kunsan National University, 558 Daehak-ro, Miryong-dong, Gunsan 54150, Korea

**Keywords:** glass fiber, foams, strength, structural design, finite element analysis, test

## Abstract

In this work, structural design and manufacturing of sandwich composite floor for automobile was performed. The tensile and compression strength of specimen were investigated. Based on this, structural design of floor board was performed. The sandwich composite floor board are subject to payload. The maximum load was analyzed in consideration of the safety factor. The structural design and analysis were performed in consideration of applied load. The finite element analysis method was applied to investigate structural safety. The stress, displacement, and buckling analysis was carried out. Through the structural analysis, it was confirmed that the designed floor board structure is safety. Based on the result, the manufacturing of prototype was conducted. Finally, test and evaluation of composite floor board was performed.

## 1. Introduction

The sandwich composite structures have been applied for internal structure of vehicles. It is due to the weight reduction which is an advantage of composite. In addition, there is an advantage that even its strength is more improved than metal structure. Recently, many composite materials have been applied also to automotive structures for weight reduction. In this work, structural design and manufacturing of floor board of automotive was performed using glass fiber composite material.

As a result of analyzing the previous study results related to sandwich composite structures, it was carried out a study in which sandwich composite structure was applied to the internal structure floor of a small-scale WIG craft’s fuselage [1]. Additionally, there was a study on post-damage repair techniques of the sandwich composite structure applied to aircraft airframe [2].

Perfetto et al. conducted study on drop test simulation and validation of a full composite fuselage section of a regional aircraft. In this work, a finite element analysis of composite fuselage of a regional aircraft was performed. The vertical drop test was conducted [3].

A. Riccio et al. performed investigation on the crashworthiness behavior of a composite fuselage sub-floor component. In this work, the crashworthiness of the floor subsection of the cargo area of a civil aircraft is investigated [4]. In addition, Riccio et al. studied experimental and numerical investigation on the crashworthiness of a composite fuselage sub-floor support system [5].

Django Mathijsen carried out a study on safety of modern aircraft with carbon fiber composite fuselages in a survivable crash [6].

Changduk Kong et al. performed a compressive strength test for the composite materials applied to aircraft structure to evaluate mechanical material properties [7].

Jianfeng Wang et al. studied the effects of core thickness and density on the laminate material properties by three-point bending and panel peeling tests [8].

Faidzi et al. studied the failure mechanisms and their contribution factors of current trends for metal-based sandwich panel. This study outlines the evolution of sandwich panels based on recent work and older sources, focusing on the trends concerning sandwich panel achievements and applications, core materials, core designs, and types of failure mechanism and factors, which contribute to the failure of sandwich panel [9].

Dong-Jun Kwon et al. performed the research on optimized epoxy foam interface of CFRP/Epoxy Foam/CFRP sandwich composites for improving compressive and impact properties [10].

Cheng-Xu Ren et al. carried out the experimental study on the quasi-static compression behavior of multilayer aluminum foam sandwich structure [11]. In this work, the influence of aluminum foam (AF) core density, stacking number, and the interlayer plates in multilayer sandwich panels structures were discussed extensively.

Garam Kim et al. investigate the effects of fluid intrusion on Nomex honeycomb sandwich structures with carbon fiber face sheets [12]. The authors focused on the lasting effect on the sandwich structure when aircraft fluids ingress into the structure.

Hesham Tuwair et al. conducted the testing and evaluation of full-scale fiber-reinforced polymer bridge deck panels incorporating a polyurethane foam core [13].

Vinayak Kallannavar et al. performed the study on effect of temperature and moisture on free vibration characteristics of skew-laminated hybrid composite and sandwich plates [14].

In the automotive structure field, there have been insufficient studies of applying sandwich composite structures. As a result of analyzing previous studies, a variety of sandwich composite structures have been applied to aircraft, but it is investigated that they are not diffused yet in the automotive field.

In this study, it was carried out a design and analysis on the internal floor board of the load-applied vehicles. The structural analysis was conducted with the finite element analysis method after analyzing the applied load. In this work, the prototype was manufactured using glass fiber after investigation on structural analysis of automotive floor.

## 2. Specification for Structural Design

The target structure in this study is a floor board of the automobile components in which a sandwich composite structure is used. The structural design load was analyzed as two cases. The first case is the one where only the loading items are loaded, which is the one where a load of 44.1 kN is applied. The second case is the one where a cart reaches safely on the metal channel-shaped structure, which is the one where a load of 9.8 kN (a weight of the cart and mounting system) is applied.

**Figure 1:**
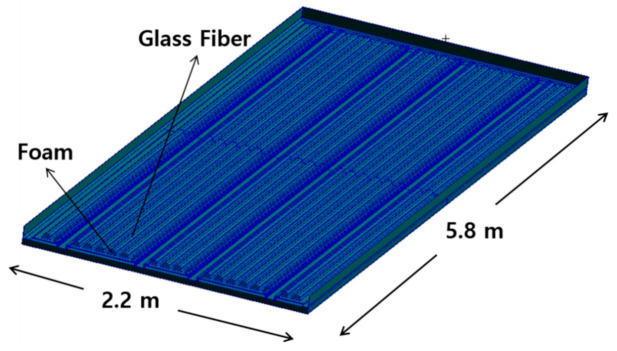
The designed floor structure for automotive.

**Figure 2:**
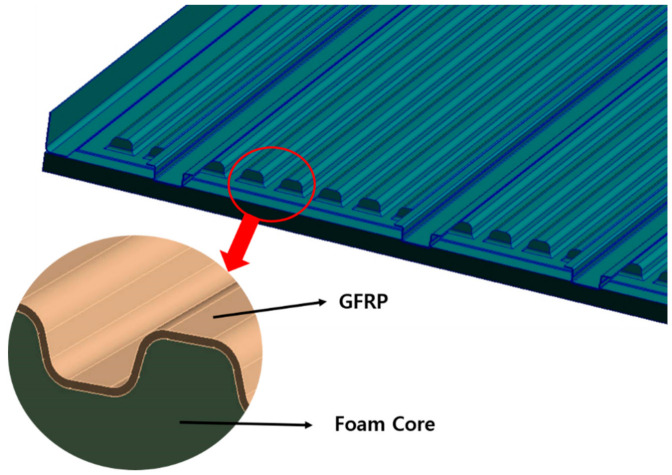
Detailed structural configuration of floor.

The material applied to the structural design is the sandwich structure in which glass fiber face sheet and foam core are adopted. The mechanical properties of glass fiber and foam core was investigated through a specimen test. The high strength steel structures were applied to another part. The sandwich composite structure was designed considering on bonding of steel and composite. The face sheet and core material were not damaged within the safety factor for the applied load. Figure 1 is a configuration of the designed floor board structure. Figure 2 shows detailed structural configuration of floor.

## 3. Mechanical Properties of Applied Materials

In this study, it was conducted to replace the material applied for automobile floor structure from the steel to the FRP lightweight material. The characteristics of the ±25 °C environmental condition applied to the vehicle were analyzed.

In this work, the material of glass fiber, vinyl ester resin, and urethane foam is adopted to design. The glass fiber/vinyl ester composite specimens were manufactured. In order to investigate the accuracy of the test specimen, the average value of the test results of five specimens was used.

**Figure 3:**
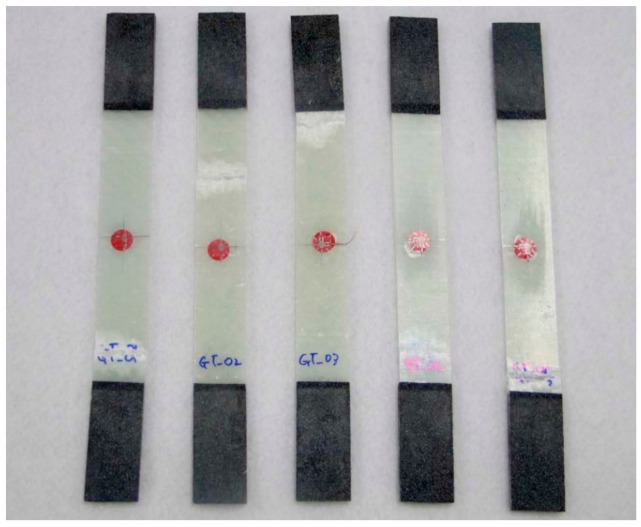
Fracture shape of tensile specimen.

**Figure 4:**
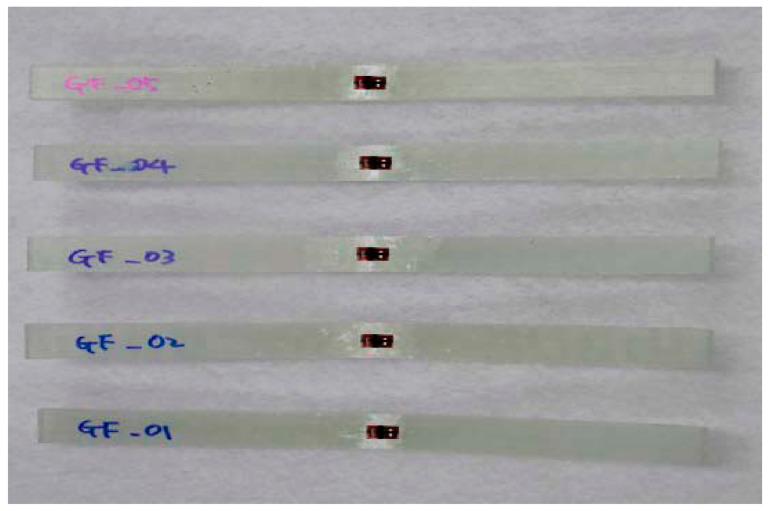
Fracture shape of compression specimen.

**Figure 5:**
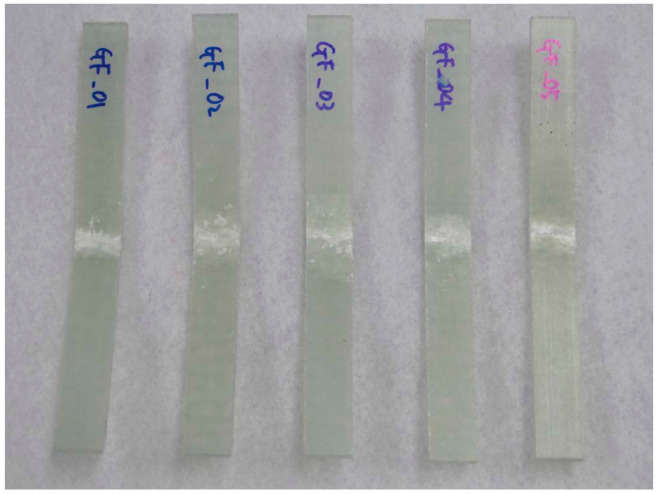
Fracture shape of flexural specimen.

**Figure 6:**
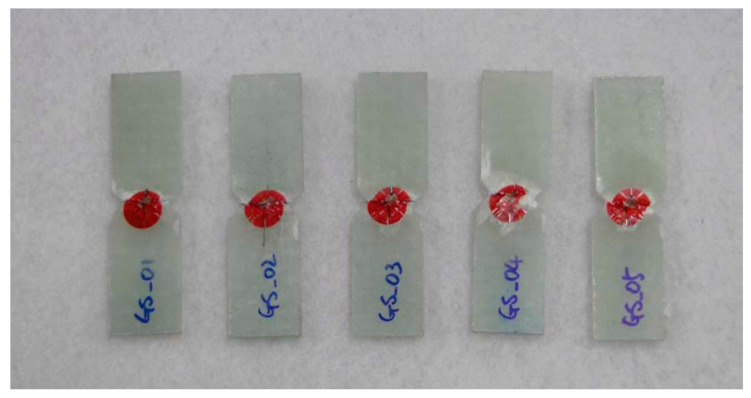
Fracture shape of shear specimen.

**Figure 7:**
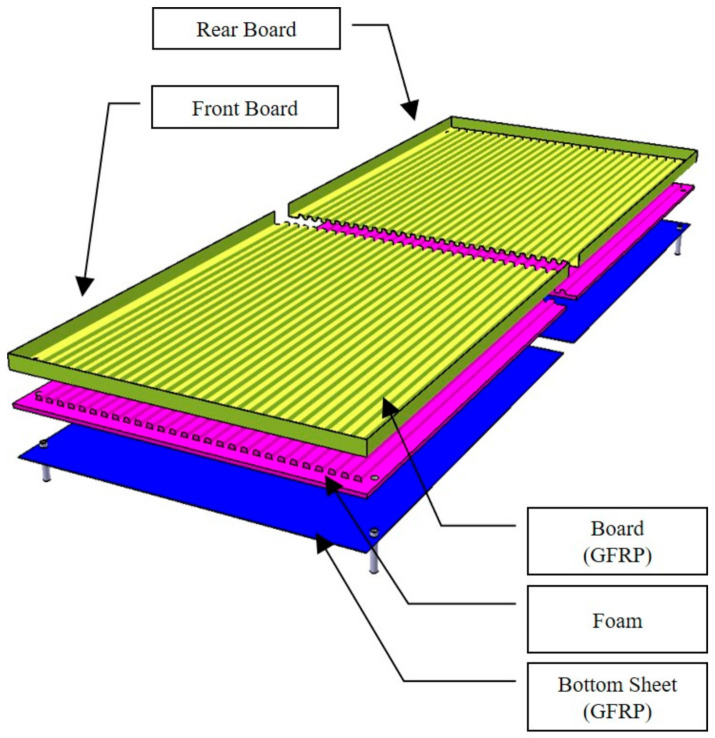
Applied material of floor structure.

**Figure 8:**
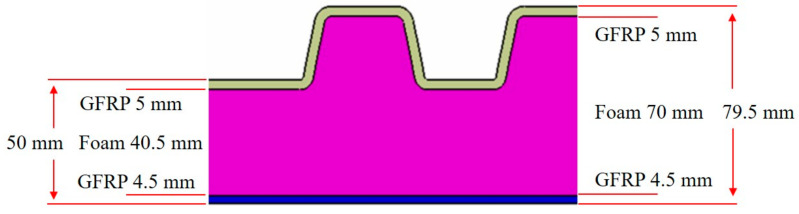
Dimension of floor cross-section.

The specimen test was performed by ASTM D3039 [15], ASTM D6641 [16], ASTM D790 [17], and ASTM D5379 [18]. The applied loading rate is 2.00 mm/min. The equipment of Instron 5582 was applied. Figure 3 and Figure 4 show the tested result of tensile and compression specimen. Figure 5 and Figure 6 show the tested result of flexural and shear specimen. The mechanical properties of each specimen test are presented in Table 1, Table 2, Table 3 and Table 4. Figure 7 shows the conceptual diagram of applied material to the floor structure. Figure 8 shows dimension of floor cross-section.

After investigation on mechanical properties of glass fiber/vinyl ester specimen, the design using glass fiber/vinyl ester and foam core was performed. The upper part of the floor is compressed by the designed load and the lower part is tensioned by the load. Therefore, the test results of tension and compression were used for each part design.

## 4. Structural Design

**Figure 9:**
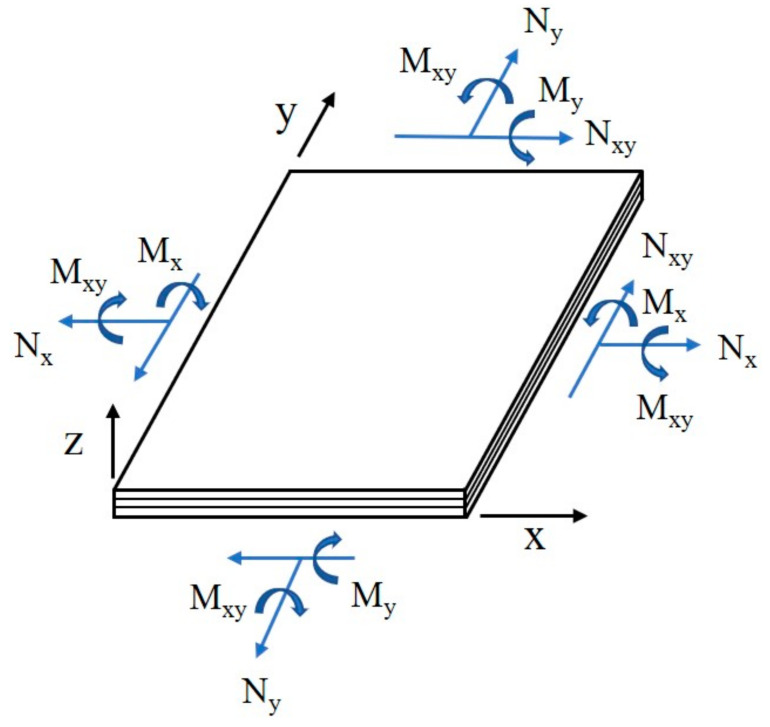
Positive system of laminate force and moment intensities at a point.

In this work, the selected target structure is a floor board of the automobile components. In the previous study, preliminary design and analysis of floor board was performed [19]. In this study, detailed structural design was performed. The structural design load of floor was defined through the design requirements. The structural design load was analyzed as two cases. The first case is the one where only the pay load is applied, which is the one where a load of 44.1 kN is applied. The second case is the one where a cart reaches safely on the metal channel-shaped structure, which is the one where a load of 9.8 kN (a weight of the cart and mounting item) is applied. The netting rule and the rule of mixture considering on composite laminate theory were used for initial structural design. The laminate netting rule, which is assuming that only fiber direction layers can provide the stiffness, i.e., no stiffness contribution from off-axis layers, is firstly applied to determine the thickness of the laminate structure. The principal stress design method is used because of the reduction in weight and well-defined load directions. The final design was performed by laminate constitutive theory. The laminate constitutive relation was presented by Equation (1).

(1) $$\left\{\begin{array}{c} N\\ M \end{array}\right\}=\left[\begin{array}{cc} A & B\\ C & D \end{array}\right]\left\{\begin{array}{c} \varepsilon^{0}\\ k \end{array}\right\}$$

$$N={\left[N_{x},N_{y},N_{xy}\;\right]}^{T}$$

$$M={\left[M_{x},M_{y},M_{xy}\;\right]}^{T}$$

$$\varepsilon^{0}={\left[\varepsilon_{x}^{0},\varepsilon_{y}^{0},\varepsilon_{z}^{0}\right]}^{T}$$

$$k={\left[k_{x},k_{y},k_{xy}\;\right]}^{T}$$

$$A=\sum {\left[\bar{Q}\right]}_{k}\left(Z_{k+1}-Z_{k}\right)$$

$$B=\frac{1}{2}\sum {\left[\bar{Q}\right]}_{k}\left(Z_{k+1}{}^{2}-Z_{k}{}^{2}\right)$$

$$D=\frac{1}{3}\sum {\left[\bar{Q}\right]}_{k}\left(Z_{k+1}{}^{3}-Z_{k}{}^{3}\right),$$

where n is loading intensities, m is moment intensities, $\varepsilon^{0}$ is membrane intensities, k is bending intensities, $\bar{Q}$ is reduced transformed stiffness matrix in the x-y axes, $Z_{k}$ is thickness-direction coordinate from middle plane of the laminate for value at bottom of layer thickness. $Z_{k+1}$ is thickness-direction coordinate from middle plane of the laminate for value at top of layer thickness. The positive system of force and moment intensities action at a point on the laminate mid-plane is shown in Figure 9.

The floor thickness can be sized by the stress and buckling strength. The stress of floor was checked by following equations considering uniform load [20].

(2) $$\sigma_{x}=-\frac{P_{x}}{h}+\frac{M_{x}\cdot z}{h^{3}/12}$$

(3) $$\sigma_{y}=-\frac{P_{y}}{h}+\frac{M_{y}\cdot z}{h^{3}/12}$$

(4) $$\tau_{xy}=\frac{P_{xy}}{h}-\frac{M_{xy}\cdot z}{h^{3}/12}$$

(5) $$\tau_{xz}=-\frac{3Q_{x}}{2h}\left[1-{\left(\frac{z}{h/2}\right)}^{2}\right]$$

(6) $$\tau_{yz}=-\frac{3Q_{y}}{2h}\left[1-{\left(\frac{z}{\frac{h}{2}}\right)}^{2}\right],$$

where σ*_x_* and σ*_y_* are normal stresses in x and y directions, τ is shear stress. *P* is in plane forces, *M_x_* and *M_y_* are bending moments per unit length on surface normal to *x* and *y* axes, *M_xy_* is twisting moment per unit length, and h is thickness. The buckling was checked by following equations. Where, k is buckling coefficient, L_x_ is x directional length of floor board, L_y_ is y directional length of floor, m = 1 (for β ≤ √2), m = 2 (for √2 ≤ β ≤ √6), m = 3 (for √6 ≤ β ≤ √12), and m = 4 (for √12 ≤ β ≤ √20). The final structural design result is shown in Figure 8. After structural design, structural analysis was performed. In order to confirm the structural safety of the structural design, structural analysis was performed using finite element analysis method. The ANSYS FEM solver was used.

(7) $$P_{cr}=kP$$

(8) $$k={\left(\frac{\beta }{m}+\frac{m}{\beta }\right)}^{2}$$

(9) $$\beta =\frac{L_{x}}{L_{y}}$$

## 5. Structural Analysis

**Figure 10:**
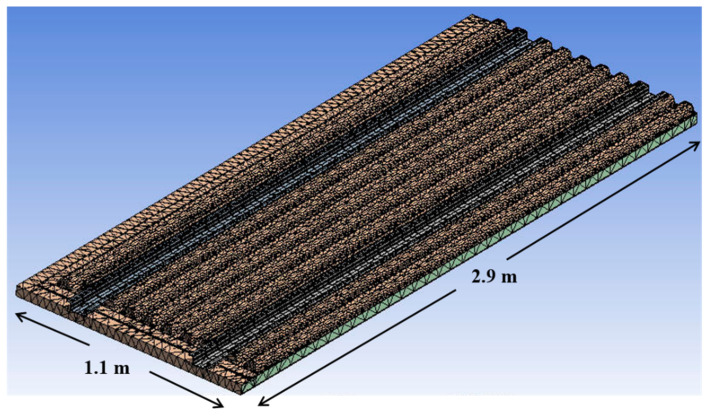
Finite element modeling result.

The finite element modeling result for performing structural analysis in this study is presented in Figure 10. The number of total elements modeled for structural analysis is 64,495 elements. The GFRP laminate structure was modelled using 2-D shell element. The foam core structure was modelled using 4 node and 3-D tetrahedral solid element. Because the whole structure was the symmetric construction, it was modeled as 1/4 to perform structural analysis. For the boundary condition, a fixed boundary condition was applied to four outer sides. Structural analysis was performed for a total of two loading conditions. For structural analysis, stress, displacement, and buckling analysis was performed to evaluate the final structural safety. The ANSYS software solver for finite element analysis was applied.

**Figure 11:**
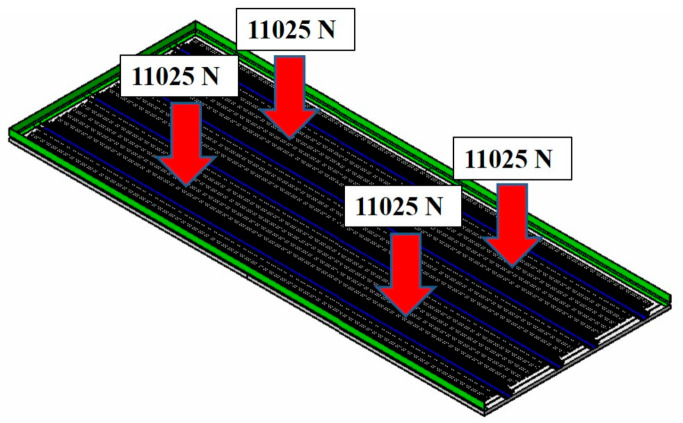
Load case 1 for structural analysis.

The first case is the one where only the payloads are applied. The structural analysis load is a case where a load of 4500 kg (44.1 kN) is applied to the whole, in which a distributed load was applied to a quarter area. Therefore, 11.025 kN was applied for the load. The conceptual diagram for load case 1 is presented in Figure 11.

**Figure 12:**
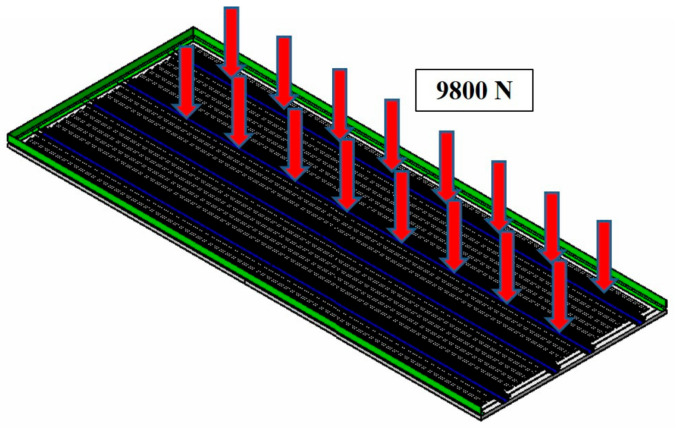
Load case 2 for structural analysis.

The structural analysis load for the second case is the one in which a cart reaches safely on the metal channel-shaped structure, and the weight of the cart and mounting structure was applied as 1000 kg (9.8 kN). The conceptual diagram for load case 2 is presented in Figure 12.

**Figure 13:**
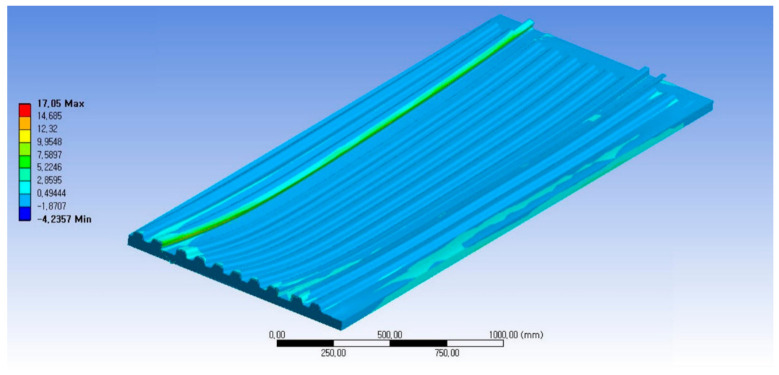
Stress analysis result of load case 1-Maximum principal stress 17.05 MPa.

As a result of structural analysis for the case where the whole load was applied to the automotive floor structure, the maximum stress was tensile stress of 17.05 MPa and compressive stress of 4.23 MPa, which was examined to be safe enough. The displacement analysis result was examined as 0.41 mm in the central part, so the displacement was also examined to be safe enough when it was combined with the vehicle. As a result of buckling analysis, the buckling load factor was high, which was examined as a structure being stable enough against buckling. The buckling analysis was performed first mode and second mode, and it was confirmed to be a sufficiently stable structure in the first mode analysis. Figure 13 shows the stress analysis result of load case 1.

**Figure 14:**
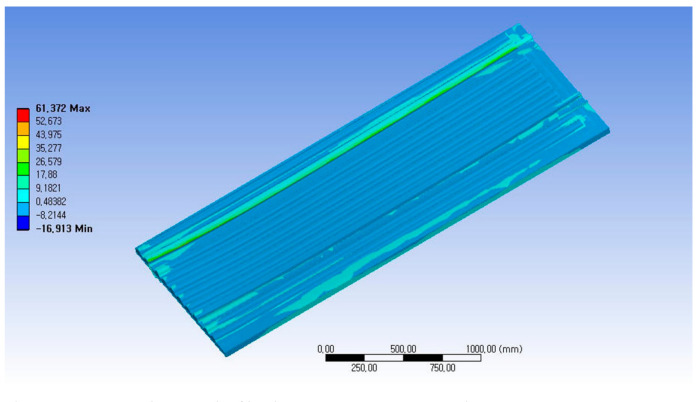
Stress analysis result of load case 2-Maximum principal stress 61.37 MPa.

As a result of structural analysis for the case where a cart reaches safely on the metal channel-shaped structure on the floor as the second case of the load application conditions, the maximum stress was tensile stress of 61.37 MPa and compressive stress of 16.91 MPa, which was examined to be safe enough. The displacement analysis result was examined as 0.91 mm in the central part, so the displacement was also confirmed to be safe enough when it was combined with the vehicle. As a result of buckling analysis, the buckling problem was examined as a structure being stable enough against buckling. Figure 14 shows the stress analysis result of load case 2.

In this study, as a result of structural analysis for the automobile floor structure in which sandwich composite and steel structures were combined, it was examined that both of two loading conditions were safe, so the design result was confirmed to be valid enough.

## 6. Manufacturing and Test of Prototype

In this study, a prototype was manufactured by reflecting the structural analysis results. RTM method was applied to the manufacturing of prototype. First, after investigation on the structural configuration, a master model was manufactured. The manufacturing of master model was performed. Considering on the positions of resin inlet and outlet, the manufacturing of molds was performed to make the prototype.

**Figure 15:**
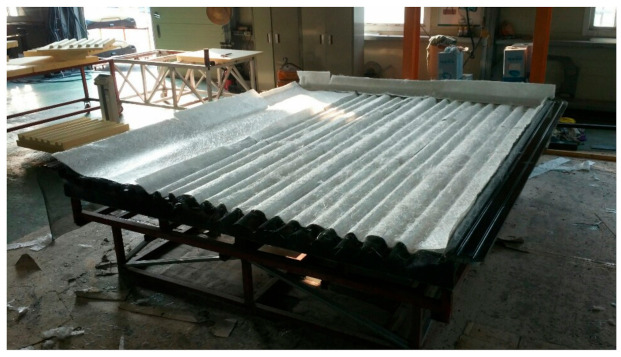
Lay-up glass fiber on mold.

**Figure 16:**
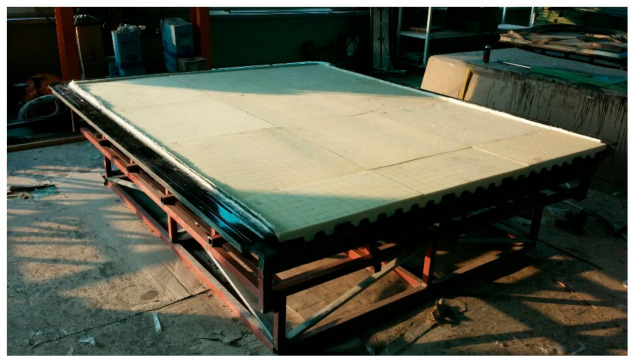
Application of foam core for sandwich composite structure.

**Figure 17:**
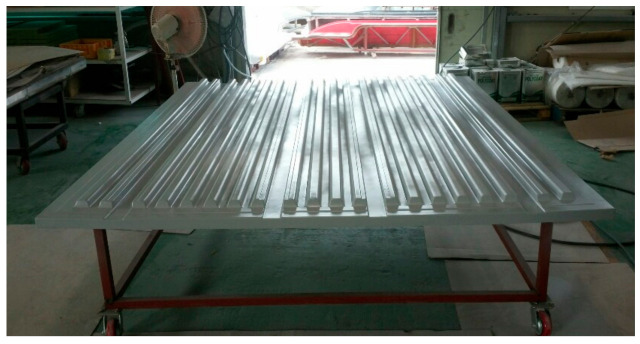
Final manufactured prototype.

After applying a gel coat to the mold, glass fibers were laminated. Figure 15 shows laminated glass fiber on the mold. After applying the foam core, the resin was injected. Figure 16 shows application of foam core for sandwich composite structure. The final prototype was manufactured using the RTM method. The final manufactured prototype is shown in Figure 17.

In this work, the structural test was performed. The manufactured prototype floor board was set on the test rig and tested by static load, and strains and deflections of the floor were measured. As a load for the static test, the designed load was applied. According to the static load test evaluation, structural safety was confirmed. The prototype floor was set on the test rig and loaded by loading system. Table 5 shows structural test results. 

In this study, the results of previous studies were investigated and reflected. Jakub Flodr et al. performed the study on experiment and numerical modeling suspended ceiling with identification of working diagram material [21]. Therefore, the literature review was conducted in this study. The comparison method of experiment and numerical modeling was reflected in this work.

In this study, the weight of the manufactured floor was measured and evaluated to compare with the existing steel structural product. The compared result is presented in Table 6. The case of applying sandwich composite material achieved weight lightening of 26% compared to steel structure.

## 7. Conclusions

In this study, structural design and analysis of the sandwich composite structure applied to automobile floor board was performed. The sandwich composite floor board is a structure that supports the payload. The maximum applied load was analyzed to apply the safety factor and perform structural design and analysis. After investigation on mechanical properties of glass fiber/vinyl ester composite, structure design of floor was performed. 

For the structural analysis, the finite element analysis method was applied to perform the stress, displacement, and buckling analysis. As a result of structural analysis for the case where the whole load was applied and the one where the load was locally concentrated, both were confirmed to be a safe structure through the stress and displacement analysis. In addition, as a result of examining the buckling analysis result thoroughly because a wide plane-shaped structure is vulnerable to buckling, it was confirmed to be a sufficiently stable structure also against buckling.

In this work, after investigation on structural design result, the prototype floor was manufactured using glass/vinyl ester and foam core sandwich composite. In order to manufacture the prototype, the RTM method is adopted. Finally, it is confirmed that the designed prototype is acceptable for structural safety and stability.

## Figures and Tables

**Table 1:** Mechanical properties of tensile specimen.

Specimen No.	Tensile Strength (MPa)	Deviation (Tensile Strength)	Modulus (GPa)	Poisson Ratio
GFRP-1T	308.50	−9.3	17.80	0.14
GFRP-2T	319.84	+2.0	22.06	0.22
GFRP-3T	325.59	+7.7	19.82	0.16
GFRP-4T	312.45	−5.3	20.11	0.18
GFRP-5T	322.67	+4.8	20.13	0.19
Average	317.81	–	19.98	0.18

**Table 2:** Mechanical properties of compression specimen.

Specimen No.	Compressive Strength (MPa)	Deviation (Compressive Strength)	Modulus (GPa)
GFRP-1C	304.59	−7.1	18.12
GFRP-2C	312.45	+0.7	18.37
GFRP-3C	318.48	+6.7	18.76
GFRP-4C	307.54	−4.1	18.24
GFRP-5C	315.50	+3.7	18.51
Average	311.71	–	18.40

**Table 3:** Mechanical properties of flexural specimen.

Specimen No.	Flexural Strength (MPa)	Deviation (Flexural Strength)	Modulus (GPa)
GFRP-1F	420.16	+11.2	19.80
GFRP-2F	421.96	+13.0	19.87
GFRP-3F	414.09	+5.2	19.99
GFRP-4F	404.37	−4.5	19.89
GFRP-5F	383.80	−25.0	19.17
Average	408.87	–	19.75

**Table 4:** Mechanical properties of shear specimen.

Specimen No.	Shear Strength (MPa)	Deviation (Shear Strength)	Modulus (GPa)
GFRP-1S	107.52	+1.1	4.24
GFRP-2S	104.96	−1.5	3.73
GFRP-3S	103.96	−2.5	4.23
GFRP-4S	112.49	+6.0	4.21
GFRP-5S	103.27	−3.2	4.05
Average	106.43	–	4.09

**Table 5:** Structural test results.

Displacement	Test Result	Analysis Result
Central part	0.33 mm	0.41 mm

**Table 6:** Comparison weight between GFRP and steel floor.

**Weight**	**Sandwich Composite Structure (kg)**	**Steel Structure (kg)**
	273.5	369.8

## Data Availability

The data presented in this study are available on reasonable request from the corresponding author.

## Abbreviations

N	loading intensities
M	moment intensities
$\varepsilon^{0}$	membrane intensities
k	bending intensities
$\bar{Q}$	reduced transformed stiffness matrix
$Z_{k}$	thickness-direction coordinate
σ*_x_*	normal stresses in x directions
σ*_y_*	normal stresses in y directions
P	plane forces
*M_xy_*	twisting moment
L_x_	x directional length of floor board
L_y_	y directional length of floor board
